# Pas Kinase Deficiency Triggers Antioxidant Mechanisms in the Liver

**DOI:** 10.1038/s41598-018-32192-w

**Published:** 2018-09-14

**Authors:** P. Dongil, A. Pérez-García, V. Hurtado-Carneiro, C. Herrero-de-Dios, E. Blazquez, E. Alvarez, C. Sanz

**Affiliations:** 1 Department of Biochemistry and Molecular Biology, Faculty of Medicine, Complutense University of Madrid, Institute of Medical Research at the Hospital Clínico San Carlos (IdISSC), Ciudad Universitaria, s/n, 28040 Madrid, Spain; 2 Spanish Biomedical Research Centre in Diabetes and Associated Metabolic Disorders (CIBERDEM), Madrid, Spain; 3 Department of Cell Biology, Faculty of Medicine, Complutense University of Madrid, Institute of Medical Research at the Hospital Clínico San Carlos (IdISSC), Ciudad Universitaria, s/n, 28040 Madrid, Spain

## Abstract

Metabolic dysfunction in the liver is the cause of numerous pathologies, which are associated with an altered redox state. PASK (PAS Domain Kinase) is a nutrient and bioenergetic sensor. We contend that PASK could act as an oxidative stress sensor in liver and/or control the metabolic balance, playing a role in the mitochondrial homeostasis. Using PASK-deficient mice, we observed that PASK deficiency promotes antioxidant response mechanisms: a lower production of ROS/RNS under non-fasting conditions, overexpression of genes coding to ROS-detoxifying enzymes and mitochondrial fusion proteins (*MnSod Gpx, Mfn1* and *Opa1*), coactivator *Ppargc1a*, transcription factors (*Pparg* and *FoxO3a*) and deacetylase *Sirt1*. Also, under fasting conditions, PASK deficiency induced the overexpression of *Ppargc1a*, *Ppara*, *Pparg*, *FoxO3a* and *Nrf2* leading to the overexpression of genes coding to antioxidant enzymes such as MnSOD, Cu/ZnSOD, GPx, HO1 and GCLm. Additionally, inducing PINK1 involved in cell survival and mitophagy. These changes kept ROS steady levels and improved the regenerative state. We suggest a new role for PASK as a controller of oxidative stress and mitochondrial dynamics in the liver. In fact, antioxidant response is PASK dependent. PASK-targeting could therefore be a good way of reducing the oxidative stress in order to prevent or treat liver diseases.

## Introduction

The most important reactive oxygen species (ROS) such as radical superoxide, non-radical hydrogen peroxide and hydroxyl radicals, are highly reactive molecules required in cell physiology. However, the excess of these molecules leads to cellular damage in DNA, proteins, and lipids.

Food intake initiates mitochondrial oxidative phosphorylation for producing ATP (adenosine triphosphate) and supporting normal cellular function and metabolic homeostasis, although mitochondrial electron transport is also the major intracellular source of ROS or “free radicals”^1,2^. Under physiological circumstances, therefore, ROS play a positive role in the cells as a signals transducer, or as a defense against invading pathogens, by modulating the key gene expression^3^. Additionally, cells protect themselves against ROS excess by intrinsic defense systems that include the expression of various antioxidant enzymes (i.e., superoxide dismutase (SOD), catalase (CAT), and glutathione peroxidase (GPx)) for converting free radicals into oxygen and water. Besides antioxidant enzymes, there are also non-enzymatic molecules such as reduced glutathione (GSH)^4,5^.

However, an exacerbated ROS production and/or an altered antioxidant mechanism could trigger oxidative stress, being harmful and highly toxic to the cell^4,6^.

Mitochondria are sources of reactive oxygen species (ROS) and display a continuous processes of fusion and fission resulting in a mitochondrial dynamic that maintain their homeostasis in order to repair different damage^7–9^. Mitochondrial dynamic and cell stress are linked. In this way, an increase in mitochondrial fusion in response to cell stress can protect from cell death and autophagy, while oxidative stress can trigger mitochondrial fission and cell death^10,11^.

The liver has metabolic machinery for meeting energy demands during normal physiology, such as glycolysis and mitochondrial oxidative phosphorylation. This condition makes it more susceptible to oxidative stress damage. However, the liver also has powerful antioxidant mechanisms. A common characteristic of many chronic liver diseases is nearly always an increase in oxidative stress, regardless of the cause of the disorder^12^.

As food deprivation is common among living organisms, mammals have developed metabolic systems to adapt to it and maintain the energy requirements needed for vital functions. Accordingly, through glycogenolysis and gluconeogenesis the liver plays a key role in maintaining whole-body energy homeostasis and glucose during fasting. Following prolonged fasting, muscle protein degradation occurs to send amino acids to the liver as another substrate for gluconeogenesis^13^. It has been reported that prolonged fasting (36 h) exerts oxidative stress, increasing hepatic free radical levels and decreasing antioxidant defenses^14,15^. Nonetheless, it has also been suggested that intermittent fasting improves oxidative stress^16–18^. As occurs in prolonged fasting, the hyperglycemic state that appears in diabetes, insulin resistance or obesity^19^ could trigger an imbalance in the cellular redox homeostasis.

Glucose availability and the homeostasis of nutrients in the cell are controlled by a nutrient or glucose sensor such as AMPK (adenosine monophosphate-activated protein kinase) and mTOR/S6K1(mammalian targets of rapamycin/Ribosomal protein S6 kinase-1). Additionally, Per-Arnt-Sim Kinase (PASK), a canonical serine/threonine kinase that contains PAS domains can sense intracellular oxygen, redox state, and also nutientes^20,21^. Furthermore, it is an emerging regulator in lipid and glucose metabolism in the liver^22^. PASK-deficient mice are protected against the development of obesity and insulin resistance induced by a high fat diet (HFD)^23–26^.

Thus, under certain physiological or pathological conditions, including hypoxia and glucose deprivation, AMPK activation enhances AMPK-mediated cellular adaptation, including the maintenance of redox homeostasis^27,28^. It seems that nutrient sensors could control the redox-state and mitochondrial function^29^. Previous studies by our group have described PASK as a critical regulator of AMPK and mTOR signaling in hypothalamus, neuroblastoma N2A cells and the liver^21,30^.

The mechanisms preventing oxidative stress remain only partially understood and require further research. Our aim here is to investigate the role PASK plays in managing hepatic oxidative stress under basal and fasting situations.

## Materials and Methods

### Isolation, culture and treatment of mouse embryonic fibroblasts

Mouse embryonic fibroblasts (MEFs) were isolated from embryos at 13.5 days of gestation. To do so, the pregnant females, wild-type (C57Bl/6J) or PASK-deficient mice (*Pask*^−/−^), were euthanized, and their fetuses were collected from the uterine branches, decapitated, and the viscera were removed. Tissue disaggregation’s were achieved by incubation in trypsin for 3–5 minutes. Cell debris was then removed by centrifugation, and MEFs were grown on a standard culture medium that contained Dulbecco’s Modified Eagle Medium (DMEM) supplemented with glucose (4.5 g/L), 15% fetal bovine serum (FBS) (Lonza) inactivated at 56 °C for 30 minutes, penicillin (100 U/mL), streptomycin (100 mg/mL) and glutamine (2 mM). The cells were maintained in a humidified atmosphere containing 5% CO_2_ at 37 °C.

The cells were treated with 2.5 mM glucose for four hours or were kept in a standard culture medium. Additionally, the cells were stimulated to a cellular stress situation by adding 200 µM H_2_O_2_ for two hours.

### Experimental animals and treatment

All the procedures involving animals were approved by the animal welfare committee of Madrid Complutense University (DC 86/609/EU) and met EU animal welfare guidelines. The animals used were 12- to 20-week-old males (25–30 g), C57BI/6 J wild-type (WT) and *Pask*^−/−^ mice crossed into C57Bl/6 J for at least 12 generations^31^. The animals were fed *ad libitum* with a standard pellet diet, and housed at a constant temperature (21 °C) on a 12-hour light-dark cycle, with lights on at 8 a.m.

Both groups of mice, *Pask*^−/−^ and WT, were kept in standard feeding conditions (*ad libitum*) (“non-fasted” or “basal” condition), or fasted for 24 or 48 hours. The mice were then decapitated and their liver was immediately frozen.

### Real-time Polymerase Chain Reaction

Liver total RNA from WT and *Pask*^−/−^ mice was extracted with TRIzol (Life Technologies, Barcelona, Spain). RNA integrity was tested with the Bioanalyzer 2100 (Agilent), and cDNA synthesis was developed using the High-capacity cDNA archive kit (Applied Biosystems), using 2 µg of RNA as template, following the manufacturer’s instructions. SYBR Green® Assay (Applied Biosystems) was used to quantify the mRNA levels by real-time quantitative RT-PCR in a 7300HT Fast Real-Time PCR System (Applied Biosystems). The details of the primers and probes are listed in Supplementary Table 1. The PCR conditions were 50 °C for 2 min, 95 °C for 10 min, followed by 40 cycles at 95 °C for 15 s, and 60 °C for 1 min. *β-actin* housekeeping gene was used for normalization, and a standard curve was previously generated in each real-time PCR assay by tenfold serial dilutions of the cDNA samples.

### Mitochondrial number and mtDNA quantification

For mitochondrial number quantification, the liver was fixed and processed properly for electron microscopy (see Supplementary Fig. S1 for details). The mitochondria were visualized by a transmission electronic microscope (JEOL JEM 1010) and micrographs were taken at 6 K magnification. Mitochondrial counts were performance at least from 7 different hepatocytes per genotype.

For mtDNA quantification, liver total DNA from WT and *Pask*^−/−^ mice was extracted in a buffer containing 10% Chelex 100 resin (Bio-Rad) and digested with Proteinase K (10 mg/mL) for 1 hour, at 55 °C. RT-PCR was performed from total DNA using primers to detect mitochondrial DNA coding for *12S* rRNA and nuclear DNA coding for *β-Actin* (Supplementary Table 1).

### Liver protein detection by Western Blotting

For the analysis of protein expression by western blotting, a tiny piece of frozen liver (~150 mg) was immediately lysed in a RIPA buffer (PBS, 1% NP-40, 0.5% sodium deoxycholate, 1 mM PMSF, 10 mM leupeptin, 1 mM NA_2_VO_4_, 25 mM Na_4_P_2_O_7_, 10 mM NaF) in presence of a protease inhibitor cocktail (Roche Diagnostics, Mannheim, Germany). The tissues were then immediately exposed to microwave irradiation for 5 s, and then homogenized^32^. After transference to a PVDF membrane (Immun-Blot® PVDF, Bio-Rad), specific proteins were detected by western blotting using the antibodies described (Supplementary Table 2), followed by incubation with the secondary antibodies bound to HRP. Stain-Free Imaging Technology was used as loading control (Mini PROTEAN^®^ TGX Stain-Free™ Gels, Bio-Rad)^33^, activating the membranes by UV exposure for 2 min using a Bio-Rad ChemiDoc MP System. Finally, the blots were quantified using Quantity One software (Bio-Rad, GS-800 Densitometer). All the western blot data were normalized by total protein visualized by the Stain-Free Imaging Technology developed by Bio-Rad. The representative membranes of the main figures are in Supplementary Figs S2 and S3.

### Immunohistochemistry

A liver sample was fixed in 4% Paraformaldehyde at 4 °C for one day, cryoprotected in 30% sucrose, frozen and sectioned at 20 µm. Protein detection was performed as described^34^. Briefly, the sections were washed in and permeabilized for 20 min with PBS-0.4% (v/v) Triton X-100. Nonspecific antibody binding sites were blocked by incubation with a blocking solution (PBS, 10% goat serum, 0.1% Triton X-100) for one hour. The liver sections were then incubated overnight (4 °C) with Rabbit anti-SIRT1 antibody diluted 1:100 in blocking solution. For fluorescence detection was used, Alexa Fluor® 488 coupled to an anti-rabbit secondary antibody (GeneTex, Inc., San Antonio, CA, USA) diluted 1:200 in a blocking solution. The nucleus was stained by adding 1 µg/ml of 4,6-diamidino-2-phenylindole (DAPI) in PBS. Finally, the liver sections were mounted on a Fluoromount G mounting medium (EMS, Hatfield, PA, USA). The images were taken with a TCS SP2 confocal laser microscopy system (Leica Microsystems, Wetzlar, Germany) equipped with an inverted DMIRE2 Leica microscope. Confocal fluorescence images were analyzed using LCS Lite software from Leica.

### ATP Concentration

Total ATP concentration was measured using the luciferase bioassay. The bioluminescence assay is based on the reaction of ATP with recombinant firefly luciferase (Sigma-Aldrich, Saint Louis, MO, USA) and its substrate luciferin (Sigma).

Cells or tissue were lysed in 20 μl of 1.35 M perchloric acid and then neutralized with 15 μl of 2.8 M KHCO_3_-0.1 M Tris. Ten microliters of the supernatant were used for the luciferase reaction by adding 10 μl of luciferase and 100 μl luciferin. Luminescence was determined in a luminometer (BG-P luminometer; GEM Biomedical, Hamden, CT). The results were normalized by total protein and expressed them all as a percentage.

### Measurement of oxygen reactive species

Oxygen reactive species (ROS) in MEFs, were measured through a fluorescence assay, using DCFH-DiOxyQ. The cells were treated with a medium containing 2.5 mM glucose and 200 µM hydrogen peroxide for two hours. Cell pellets were incubated in 100 μl of reagent 40 μM 2′,7′-diclorodihydrofluorescein diacetate (H_2_DCFDA) (Molecular Probes/Invitrogen Life Technologies) for one hour. The measurement was carried out in a fluorescence plate reader (Thermo Fisher Scientific) at 480 nm excitation/530 nm emission. The results were normalized with a MTT (3-(4,5-dimethylthiazol-2-yl)-2,5-diphenyltetrazol bromide) measurement.

Total reactive species levels (ROS/RNS) in the liver were measured using the OxiSelect *in vitro* ROS/RNS Assay Kit Green Fluorescence (Cell Biolabs, INC.), following the manufacturer’s instructions. The ROS values were extrapolated from a standard curve, built with tenfold serial dilutions of 1 mM DCF (2′,7′-dichlorodihydrofluorescein).

### Determination of Citrate synthase (CS) and Manganese superoxide dismutase (MnSOD) activities

Citrate synthase (CS) activity of liver samples under the conditions indicated was measured using the Citrate Synthase Activity Assay Kit from Sigma (MAK193), following manufacturer’s instructions. CS activity was determined using a coupled enzyme reaction, resulting in a colorimetric (412 nm) product proportional to the enzymatic activity. The reaction was measured every 5 min for 40 min at 25°, considering the penultimate reading the final result. CS activity was represented as a percentage. To measure the liver cytosolic SOD activity, a commercially available kit (STA-340, Cell Biolabs, INC.) was used according to the manufacturer’s protocol. Absorbance was measured at 490 nm after 1 h incubation at 37 °C. SOD activity was represented as a function of inhibition percentage.

### Statistical analyses

Data are presented as means ± SEM. For experiments with multiple comparisons, the differences between groups were first tested with ANOVA, followed by pairwise t-test comparisons with Tukey’s post hoc test, to determine the differences across groups. Data were considered statistically significant at P ≤ 0.05. Statistical analyses were performed using GraphPad Prism software.

## Results

### Lower *in vivo* content of ATP and ROS/RNS in the liver of non-fasted *Pask*^−/−^ mice

The production of ATP and ROS is part of the normal oxidative metabolism that is finely regulated by a network that includes intra- and inter-cellular mechanisms and could be specific to certain organs or tissues. We therefore studied ATP and ROS/RNS liver content *in vivo* in WT and PASK-deficient mice *(Pask*^−/−^).

**Figure 1:**
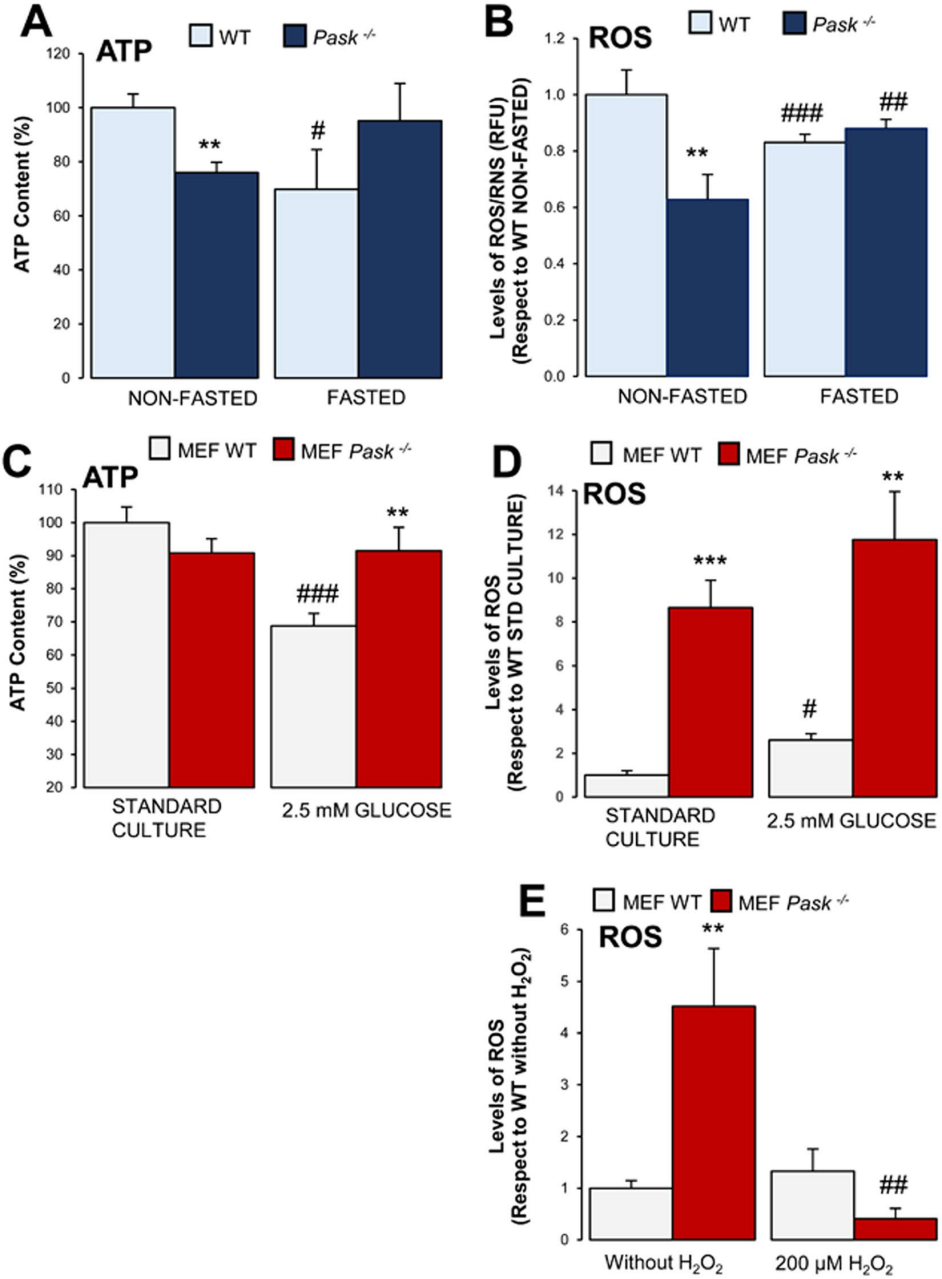
Effects of glucose and PASK deficiency on ATP and ROS/RNS content, in liver and mouse embryonic fibroblasts (MEFs). (**A**) ATP content and (**B**) levels of ROS/RNS were measured under non-fasted (NON-FASTED) and fasted 48 h (FASTED) conditions in livers from wild-type (WT) and PASK-deficient (*Pask*^−/−^) mice. (**C**) ATP content and (**D,E**) levels of ROS were measured in a standard culture (STANDARD CULTURE) and a low glucose culture (2.5 mM GLUCOSE) in MEFs from wild-type (WT) and PASK-deficient (*Pask*^−/−^) mice. Cells were treated, or not, with 2.5 mM glucose for four hours (**C,D**), and in other cases, (**E**) 200 µM of hydrogen peroxide was added for two hours. The value obtained in NON-FASTED WT mice and STANDAR CULTURE MEF WT was taken as 1. The ATP results were normalized by total protein and expressed as a percentage, and ROS/RNS levels as RFU. Bar graphs in (**A,B**) represent means ± SEM; n = 4–5 animals per condition. **P < 0.01 WT *vs. Pask*^−/−^*;* ^#^*P* < 0.05, ^##^*P* < 0.01, ^###^*P* < 0.001 NON-FASTED *vs*. FASTED; (**C**–**E**) represent means ± SEM; n = 4–5 experiments developed in duplicate. **P < 0.01, ***P < 0.001 MEF WT *vs*. MEF *Pask*^−/−^; ^#^*P* < 0.05, ^##^*P* < 0.01, ^###^*P* < 0.001 STANDARD CULTURE *vs*. 2.5 mM GLUCOSE or without H_2_O_2_ vs 200 μM H_2_O_2_.

*Pask*^−/−^ mice recorded lower ATP levels than WT, under non-fasted conditions. While fasting reduced ATP levels in WT mice, *Pask*^−/−^ kept ATP at similar levels to non-fasted WT (Fig. 1A). ROS/RNS content was consistent with ATP levels in both WT and PASK-deficient mice (Fig. 1B). Lower ATP levels were found in PASK-deficient mice (non-fasted) compared to WT, while they were similar to WT basal in fasted *Pask*^−/−^ (Fig. 1A).

### PASK-deficient MEFs were better protected against ROS triggered by H_2_O_2_ in an *in vitro* model

We analyzed the response of PASK-deficient cells to oxidative stress situations. MEFs *Pask*^−/−^ were kept in a standard medium with full glucose availability, or else restricted to 2.5 mM glucose for four hours.

Our results showed that ATP levels in WT MEF cells depended on glucose availability. The cells grown in a low glucose concentration for four hours failed to generate ATP (Fig. 1C). However, ATP content remained at the same level in PASK-deficient cells, even when glucose levels decreased. This proved that PASK deficiency involved active ATP production even during glucose restriction and reached the same levels as in WT at higher glucose levels (Fig. 1C). These results indicated that PASK-deficient cells generally presented a mitochondrial function that is activated independently of glucose deprivation.

Additionally, PASK-deficient MEFs produced a greater amount of ROS than in WT, under all the conditions studied (Fig. 1D). However, when cells were exposed to a stress situation such as elevated H_2_O_2_, ROS levels were blocked in the PASK-deficient cells (Fig. 1E).

### PASK deficiency altered hepatic mitochondrial content and biogenesis

The lower ATP content reported here in the liver of PASK-deficient mice kept under standard conditions (non-fasted) could be due to low mitochondrial activity, which could most likely be attributed to a lower mitochondrial content. This hypothesis has been studied. Our data indicated that the average number of mitochondria in the hepatocytes of *Pask*^−/−^ was lower than in WT (Supplementary Fig. S1), under non-fasting or fasting conditions (31% and 87% less, respectively). We therefore tested the expression of the transcription factors and regulators of mitochondrial biogenesis.

**Figure 2:**
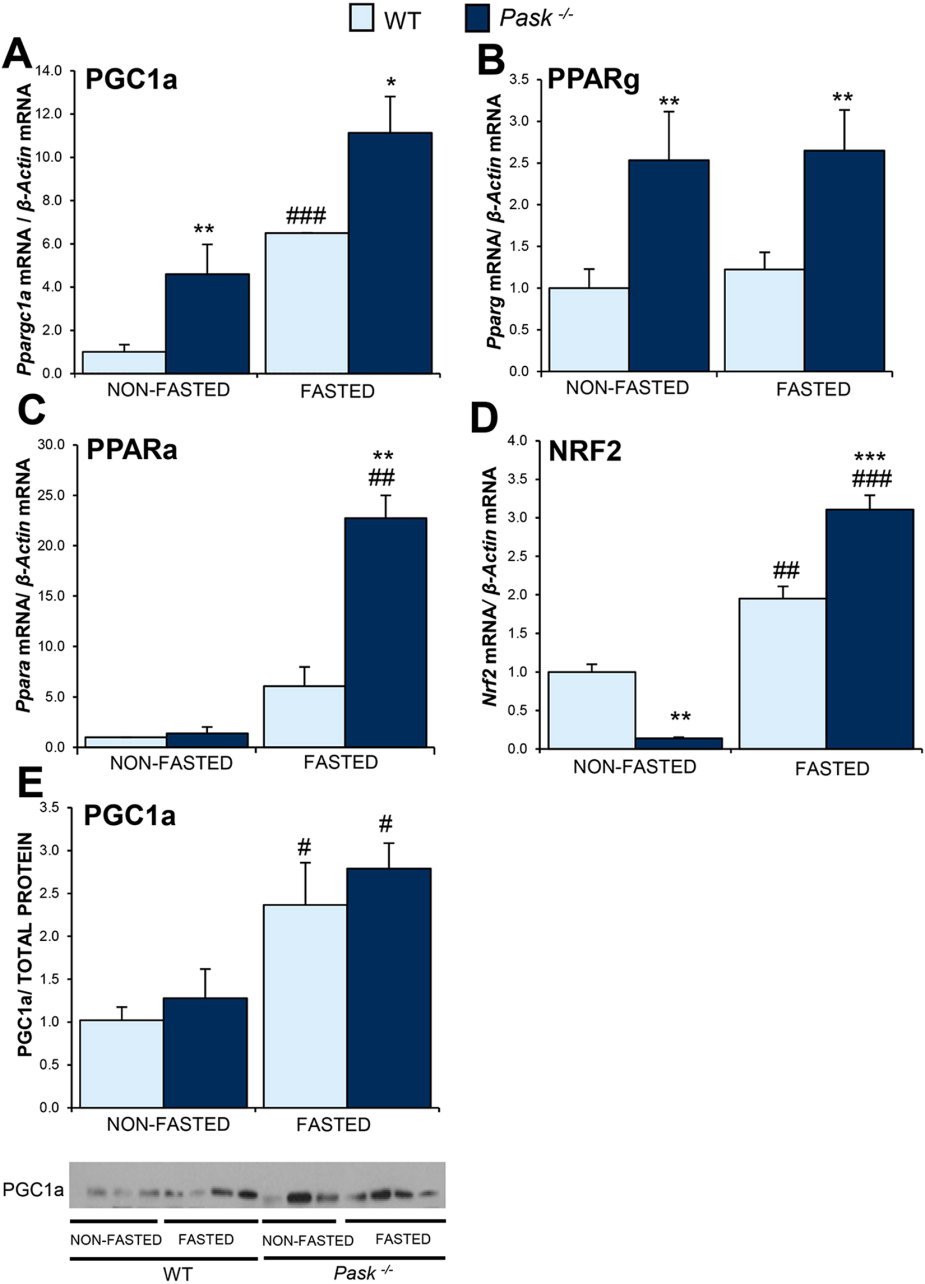
Effects of PASK deficiency on the expression of several hepatic genes involved in mitochondrial biogenesis and PGC1a protein. Quantitative real-time PCR was used to analyze the expression of genes as shown in the Supplementary Table 1. The mRNA levels were measured under non-fasted (NON-FASTED) and fasted 48 h (FASTED) condition in livers from wild-type (WT) and PASK-deficient (*Pask*^−/−^) mice. The value obtained in NON-FASTED WT mice was taken as 1. Bar graphs in (**A**–**D**) represent means ± SEM, and the levels of expression were normalized by the mRNA of *β-Actin* used as housekeeping gene; (**E**) represents means ± SEM of the densitometric values normalized by total protein detected by Stain-Free (TOTAL PROTEIN) (Supplementary Fig. S2); n = 4–5 animals per condition. **P < 0.01, ***P < 0.001 WT *vs. Pask*^−/−^; ^#^*P* < 0.01, ^##^*P* < 0.01, ^###^*P* < 0.001 NON-FASTED *vs*. FASTED.

Adapting to energy requirements under prolonged stimulus may require activation at transcriptional level. This process is controlled by several transcription factors that include nuclear respiratory factors (NRFs), peroxisome proliferator-activated receptors (PPARs), and estrogen-related receptors (ERRs). The activity of these transcription factors is modulated by transcriptional coactivators such as PGC1a (peroxisome proliferator-activated receptor gamma coactivator 1-alpha). It is known to regulate mitochondrial biogenesis and therefore we analyzed the gene expression of *Ppargc1a* encoding PGC1a and some of the genes for transcription factors and nuclear receptors involved in mitochondrial biogenesis: *Nrf2*, *Ppara*, *Pparg*. The overexpression of both *Ppargc1a* and *Pparg* was observed in PASK-deficient mice compared to WT (Fig. 2A,B), under both fasted and non-fasted conditions. However, *Ppara* maintained the same expression under basal conditions (Fig. 2C), while *Nrf2* expression decreased (Fig. 2D), but the expression of *Nrf2* was higher in fasted PASK-deficient mice compared to WT. PGC1a protein expression was detected by Western blotting, and although the level increased after fasting in both WT and PASK-deficient mice, no significant differences were observed in *Pask*^−/−^ compared to WT (Fig. 2E), as was the case with the mRNA (Fig. 2A).

### *Sirt1* expression was upregulated in non-fasted PASK-deficient mice

**Figure 3:**
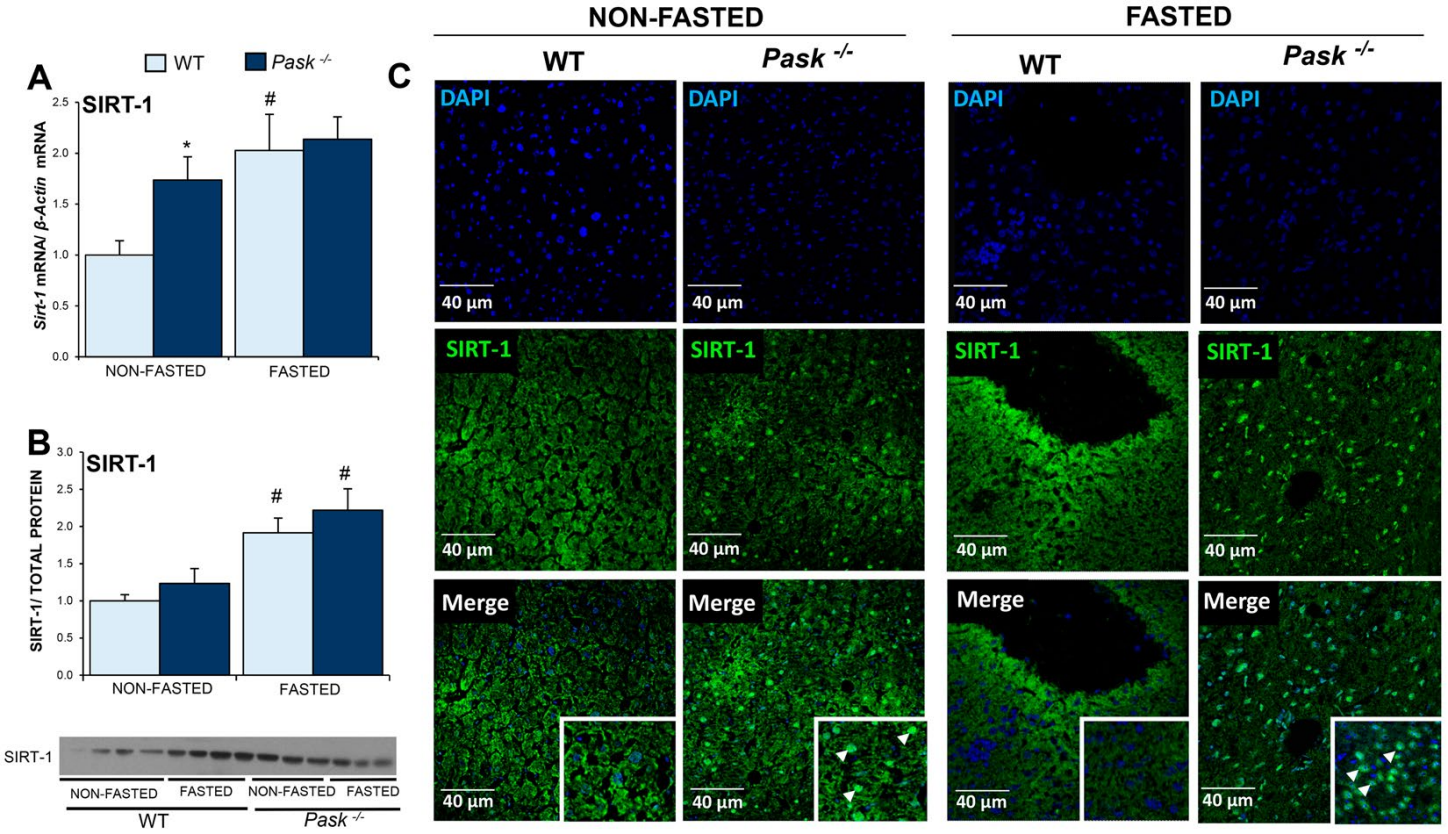
Effects of PASK deficiency on the expression of mRNA, protein, and the sub-cellular distribution of SIRT1. Quantitative real-time PCR was used to analyze the expression of *Sirt1* mRNA levels (**A**), Immunoblot analysis of SIRT1 by Western blotting (**B**) and Immunohistochemistry of SIRT1 (**C**), in livers from non-fasted (NON-FASTED), fasted 48 h (FASTED) wild-type (WT) and PASK-deficient (*Pask*^−/−^) mice. The value obtained in NON-FASTED WT mice was taken as 1. Bar graphs in (**A**) represent means ± SEM, and the levels of expression were normalized by the mRNA of *β-Actin* used as housekeeping gene; (**B**) represents means ± SEM of the densitometric values normalized by total protein detected by Stain-Free (TOTAL PROTEIN) (Supplementary Fig. S2); n = 4–5 animals per condition. *P < 0.05 WT *vs. Pask*^−/−^; ^#^*P* < 0.05, NON-FASTED *vs*. FASTED. SIRT1 sub-cellular distribution (**C**) is shown in green (Alexa 488 coupled to a secondary antibody). Nuclei were stained with DAPI (blue fluorescence). Inserts shown higher magnification. Arrows indicate SIRT1 nuclear location.

Post-transcriptional PGC1a activity is also regulated by the deacetylation exerted by SIRT1. Here, our results showed that both, the expression of *Sirt1* and protein, were upregulated by fasting in WT mice. *Sirt1* gene was overexpressed under basal conditions in PASK-deficient mice. This did not translate to higher SIRT1 protein levels either in non-fasting or fasted conditions in PASK-deficient mice compared to WT (Fig. 3A,B). Interestingly, SIRT1 sub-cellular localization shifted to mostly nuclear in PASK-deficient mice, whereas SIRT1 was mainly located in the cytosol under both conditions in WT mice (Fig. 3C).

### Mitochondrial DNA content and function were altered in PASK-deficient mice

In order to evaluate mitochondrial abundance and biogenesis in the liver from PASK-deficient mice, we analyzed hepatic mitochondrial DNA content, by the ratio of mitochondrial/nuclear DNA and some of the components of the machinery involved in different mitochondrial functions. PGC1a, other transcription factors and nuclear receptors are able to regulate the expression of mitochondrial proteins. Examples are citrate synthase (CS) (Krebs cycle enzyme) and medium-chain acyl-CoA dehydrogenase (MCAD), which catalyze the initial step of fatty acid β-oxidation. The expression of these mitochondrial enzymes and CS activity have been used to determine mitochondrial function. In addition, some genes in the electronic transport complex, such as cytochrome c, oxidase subunit IV (COX-IV) were checked.

**Figure 4:**
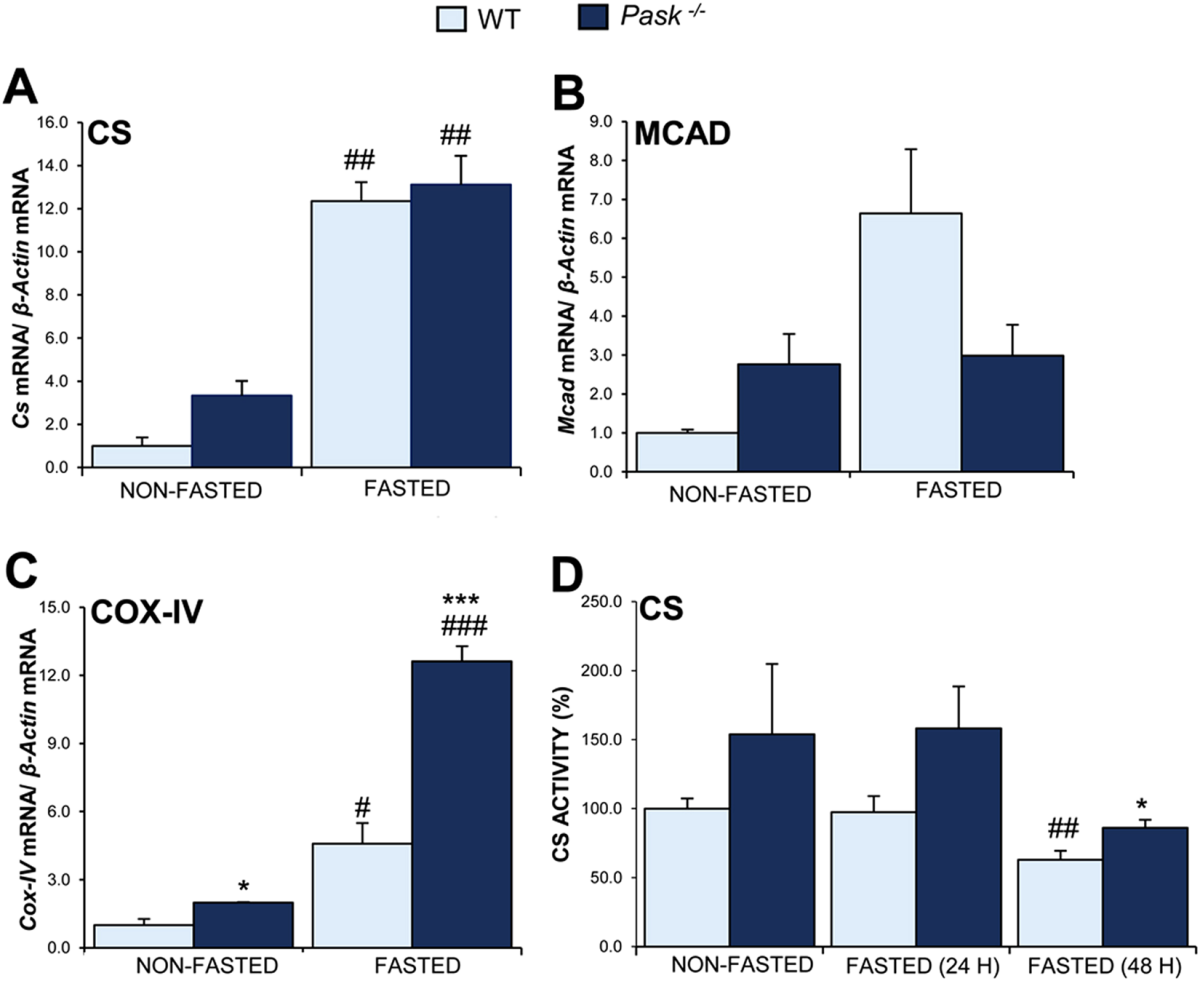
Effects of PASK deficiency on the expression and activity of some mitochondrial proteins. Quantitative real-time PCR was used to analyze the expression of *Cs* (**A**), *Mcad* (**B**) and *Cox-IV* (**C**) mRNA levels. Citrate synthase activity in liver homogenates was also measured (**D**). The results were measured under non-fasted (NON-FASTED) and fasted 48 h (FASTED) conditions in livers from wild-type (WT) and PASK-deficient (*Pask*^−/−^) mice. The mRNA levels of different genes were normalized by the mRNA of *β-Actin* used as housekeeping gene. The value obtained in NON-FASTED WT mice was taken as 1. Bar graphs represent means ± SEM; n = 4–5 animals per condition. *P < 0.05, ***P < 0.001 WT *vs. Pask*^−/−^; ^#^*P* < 0.05, ^##^*P* < 0.01 ^###^*P* < 0.001 NON-FASTED *vs*. FASTED. Citrate synthase activity was measured under non-fasted (NON-FASTED) and fasted 24 and 48 h (FASTED 24, 48 H) conditions in livers from WT and *Pask*^−/−^ mice and results were expressed as a percentage. Bar graphs represent means ± SEM; n = 4–5 animals per condition. *P < 0.05 WT *vs. Pask*^−/−^; ^##^*P* < 0.01 NON-FASTED *vs*. FASTED 48 H.

Mitochondrial DNA content was higher in PASK-deficient mice than in WT, both, under basal (non-fasted) (9.31 ± 1.25 *vs* 3.81 ± 0.03) and fasted conditions (10.30 ± 1.40 *vs*. 5.34 ± 0.14). Moreover, *Cox-IV* was overexpressed in the liver from PASK-deficient mice (Fig. 4), which had a slightly higher expression of *Cs* and *Mcad* genes, under basal conditions. All of these genes increased their expression in fasted WT mice. Under this fasting condition, PASK-deficient mice maintained a similar expression of *Cs* and *Mcad* (Fig. 4A,B), while a higher *Cox-IV* expression was observed (Fig. 4C).

Citrate synthase activity in liver homogenates was slightly higher in PASK-deficient mice, under all feeding/fasting conditions. Interestingly, CS activity remains high even in long fasting (48 h) when CS activity decreased in WT (Fig. 4D).

### PASK deficiency altered mitochondria remodeling and mitophagy processes

**Figure 5:**
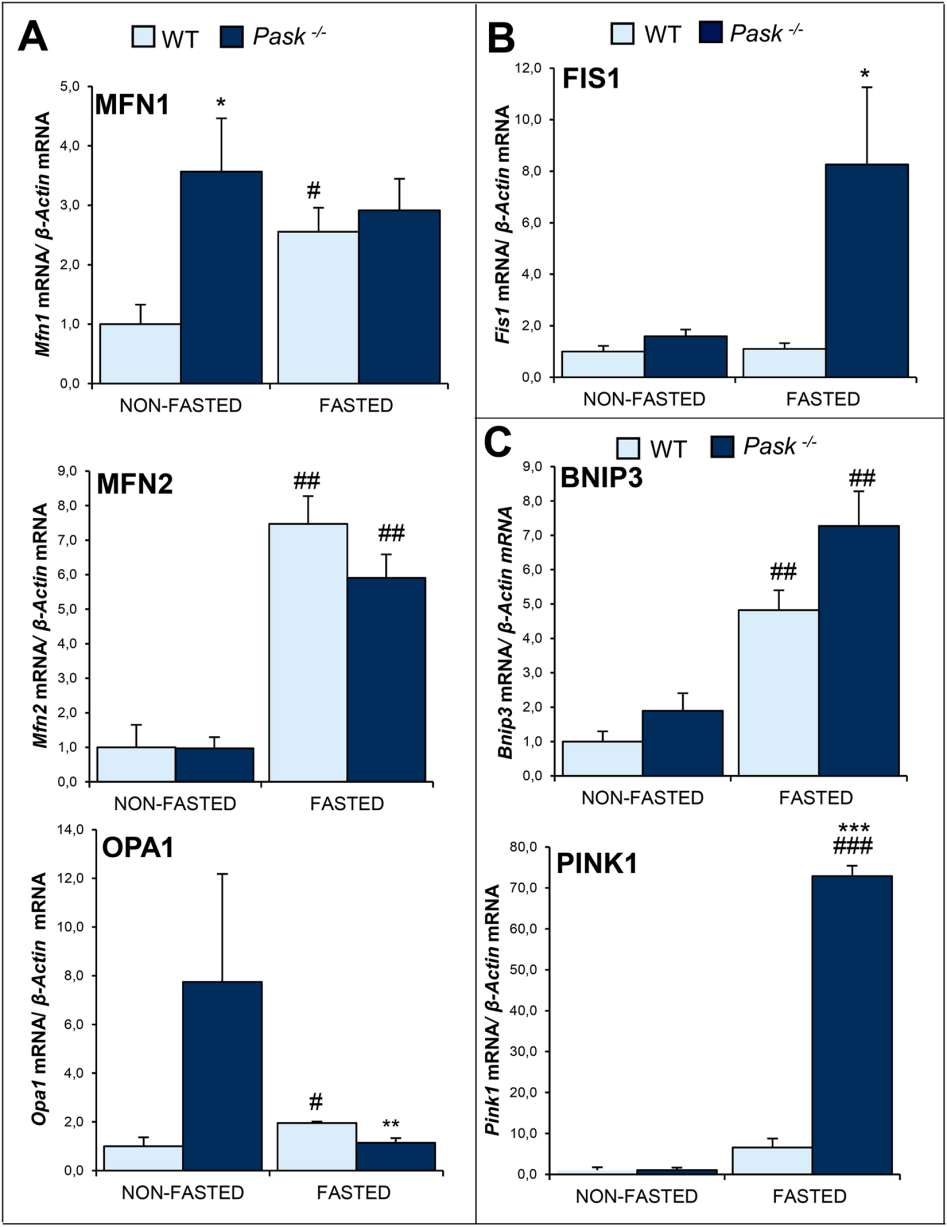
Effects of PASK deficiency on the expression of several hepatic proteins involved in mitochondria remodeling and mitophagy process. Quantitative real-time PCR was used to analyze the expression of fusion proteins *Mfn1*, *Mfn2* and *Opa1* (**A**), fission protein *Fis1* (**B**) and mitophagic proteins *Bnip3* and *Pink1* (**C**) mRNA levels. The results were measured under non-fasted (NON-FASTED) and fasted 48 h (FASTED) conditions, in livers from wild-type (WT) and PASK-deficient (*Pask*^−/−^) mice. The mRNA levels of different genes were normalized by the mRNA of *β-Actin* used as housekeeping gene. The value obtained in NON-FASTED WT mice was taken as 1. Bar graphs represent means ± SEM; n = 4–5 animals per condition. *P < 0.05, **P < 0.01, ***P < 0.001 WT *vs. Pask*^−/−^; ^#^*P* < 0.05, ^##^*P* < 0.01, ^###^*P* < 0.001 NON-FASTED *vs*. FASTED.

**Figure 6:**
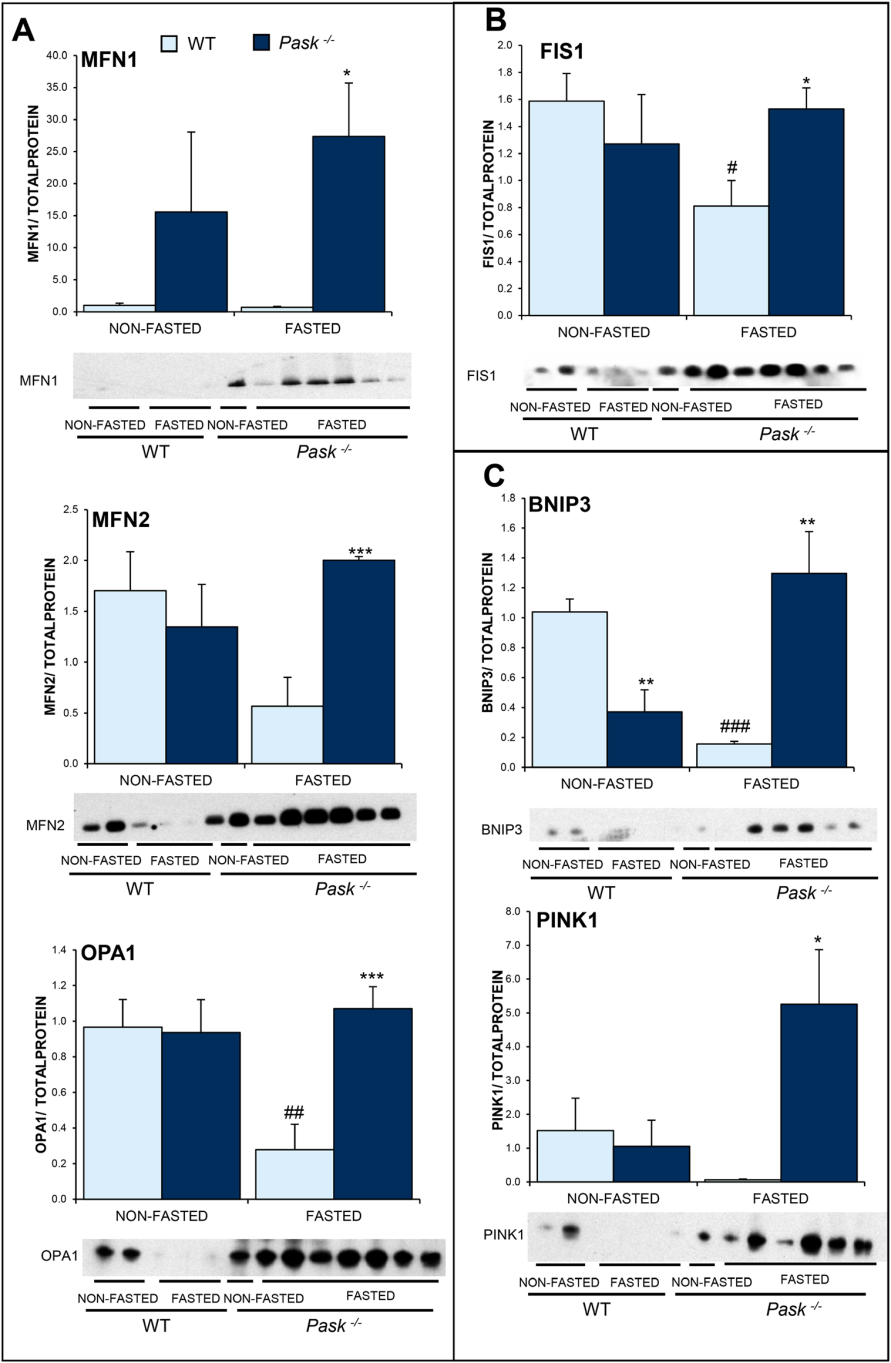
Effects of PASK deficiency on the levels of several hepatic proteins involved in mitochondrial dynamics and mitophagy. Immunoblot detection of fusion proteins MFN1, MFN2 and OPA1 (**A**), fission protein FIS1 (**B**) and mitophagic proteins BNIP3 and PINK1 (**C**), in livers from wild-type (WT) and PASK-deficient (*Pask*^−/−^) mice. Liver lysates from non-fasted (NON-FASTED) or 48 h fasted (FASTED) mice were processed for Western blot analysis. The value obtained in NON-FASTED WT mice was taken as 1. Bar graphs represent means ± SEM of the densitometric values normalized by total protein detected by Stain-Free (TOTAL PROTEIN) (Supplementary Fig. S3); n = 4–5 animals per condition. *P < 0.05, **P < 0.01, ***P < 0.001 WT *vs. Pask*^−/−^; ^#^*P* < 0.05, ^##^*P* < 0.01, ^###^*P* < 0.001 NON-FASTED *vs*. FASTED.

Mitochondrial dynamic is based on fusion and fission phenomena, and plays a key role in the maintenance of viable mitochondria. Basically, the mitochondrial functionality depends on the production of new mitochondria (biogenesis) and the elimination of non-functional ones (mitophagy). We analyzed some of the components that regulate these processes (Figs 5 and 6). Mitofusin-1 (Mfn1) and Mfn2, Optic atrophy 1 (Opa1) (all involved in mitochondrial fusion) (Figs 5A and 6A); Mitochondrial receptor protein, Fission 1 (Fis1) (Figs 5B and 6B) and BCL2 and adenovirus E1B 19-kDa-interacting protein (BNIP3), Phosphatase and tensin homolog (PTEN)-induced kinase 1 (PINK1) to study the mitophagy process (Figs 5C and 6C).

PASK deficiency upregulated the expression of genes related to mitochondrial fusion (*Mnf1* and slight *Opa1*) under non-fasting conditions when were compared to WT mice (Fig. 5A), however no differences were observed in genes of mitochondrial fission or mitophagy (Fig. 5B,C). On the contrary, PASK deficiency induced the expression of fission and mitophagic genes (specially *Fis1 and Pink1*, respectively*)* when mice were fasted for 48 h (Fig. 5B,C). It was notorious that almost all the protein levels studied were downregulated (contrary to gene expression that was upregulated) by fasting in WT mice. However, *Pask*^−/−^ mice maintained high the protein levels in that situation (Fig. 6).

### PASK deficiency induced antioxidant gene expression

The control of oxidative stress also depends on the expression levels of antioxidant enzymes. We analyzed the effect of PASK deficiency on the mRNA levels of ROS detoxification machinery: Catalase (CAT), Superoxide Dismutase (SOD: MnSOD mainly mitochondrial and Cu/ZnSOD located in cytosol) and Glutathione Peroxidase (GPx).

**Figure 7:**
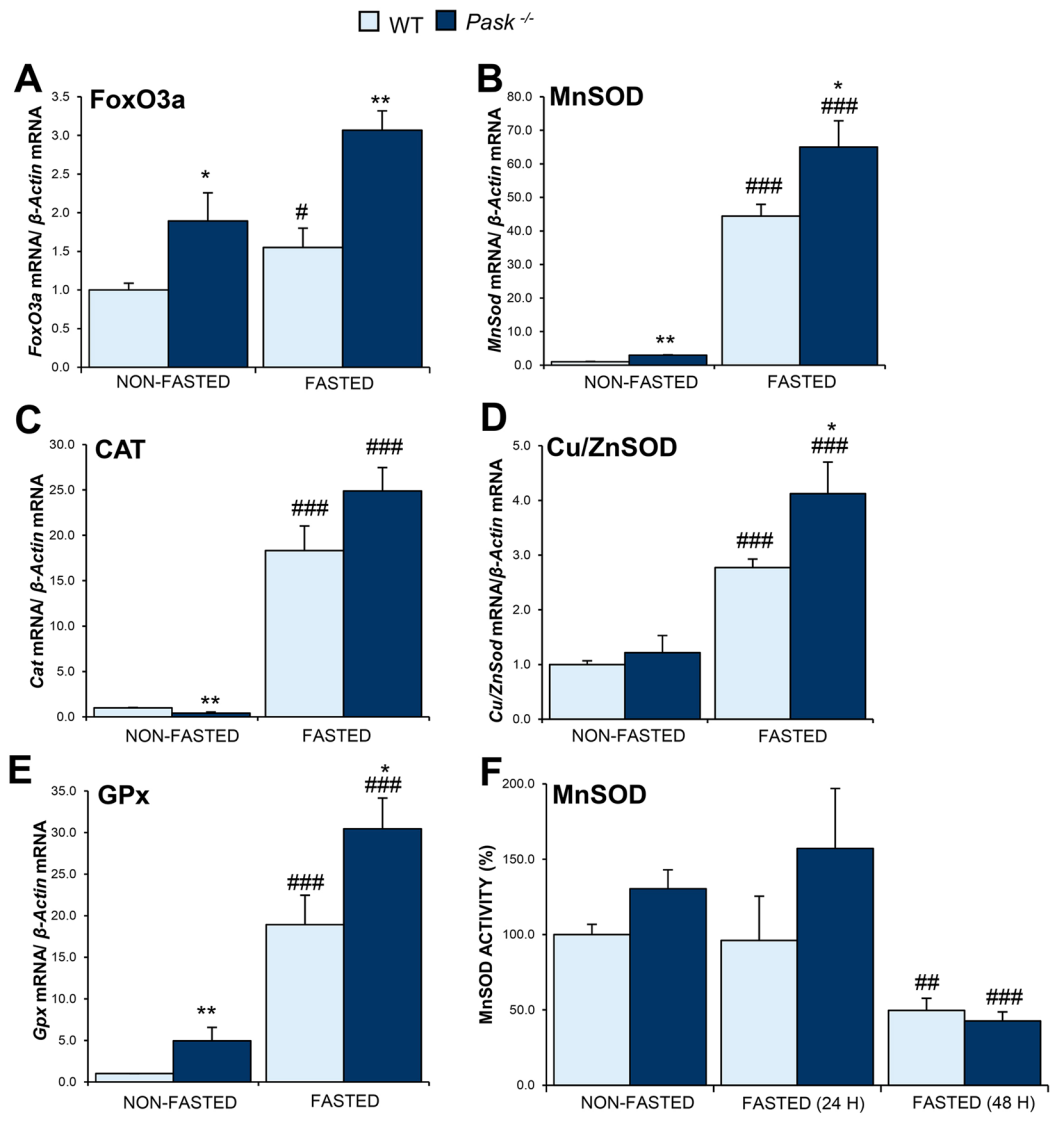
Effects of PASK deficiency on the expression and activity of several oxidative stress protective genes. Quantitative real-time PCR was used to analyze the expression of *FoxO3a* (**A**), *MnSod* (**B**), *Cat* (**C**), *Cu/ZnSod* (**D**) and *Gpx* (**E**) mRNA levels. MnSOD activity in liver homogenates was measured (**F**). The results were measured under non-fasted (NON-FASTED) and fasted 48 h (FASTED) conditions in livers from wild-type (WT) and PASK-deficient (*Pask*^−/−^) mice. The mRNA levels of different genes were normalized by the mRNA of *β-Actin* used as housekeeping gene. The value obtained in NON-FASTED WT mice was taken as 1. Bar graphs represent means ± SEM; n = 4–5 animals per condition. *P < 0.05, **P < 0.01 WT *vs. Pask*^−/−^; ^#^*P* < 0.05, ^###^*P* < 0.001 NON-FASTED *vs*. FASTED. MnSOD activity in liver homogenates was measured under non-fasted (NON-FASTED) and fasted 24 and 48 h (FASTED 24, 48 H) conditions in livers from WT and *Pask*^−/−^ mice and results were expressed as a percentage. Bar graphs represent means ± SEM; n = 4–5 animals per condition. ^##^*P* < 0.01, ^###^*P* < 0.001 NON-FASTED *vs*. FASTED 48 H.

SOD transforms the superoxide radical into either ordinary molecular oxygen (O_2_) or hydrogen peroxide (H_2_O_2_) and then CAT degrades H_2_O_2_. The expression of MnSOD and CAT is induced by the FoxO3a-SIRT1 complex (Forkhead box protein O3a-Sirtuin1). Our data showed that expression of *FoxO3a* was induced by fasting both in WT and PASK-deficient mice. Additionally, it was overexpressed under all conditions in PASK-deficient compared to WT mice (Fig. 7A). We likewise observed that fasting induced the expression of all the antioxidant enzyme genes (Fig. 7B–E). In sum, *Pask*^−/−^ mice recorded a better induction of antioxidant enzyme genes, in response to fasting (Fig. 7B–E). In the case of *MnSod* and *Gpx* (Fig. 7B,E), PASK-deficient mice had higher levels than WT in the expression of these genes also under basal conditions.

MnSOD activity in liver homogenates was slightly higher in PASK-deficient mice, under basal (non-fasting) and 24 h fasting conditions. However, 48 h fasting decreased the MnSOD activity in both, WT and PASK-deficient mice (Fig. 7F).

### PASK deficiency altered the protein levels of NRF2, GCLm and HO1

**Figure 8:**
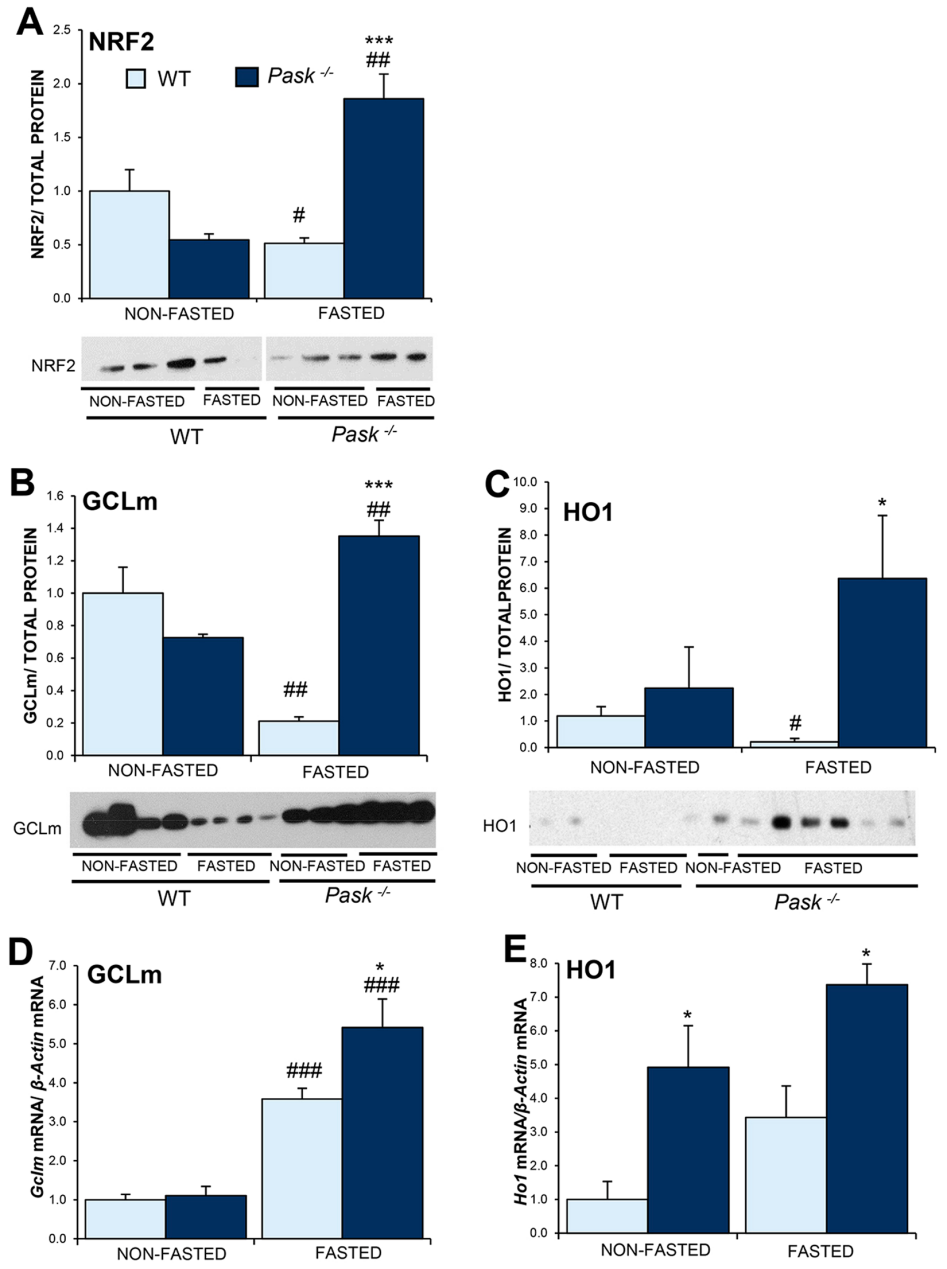
Effects of PASK deficiency on the NRF2, GCLm and HO1 proteins. Immunoblot analysis of NRF2 (**A**), GCLm (**B**) and HO1 (**C**), in livers from wild-type (WT) and PASK-deficient (*Pask*^−/−^) mice. Liver lysates under non-fasted (NON-FASTED) and fasted 48 h (FASTED) conditions were processed for Western blot analysis. Quantitative real-time PCR was used to analyze the expression of *Gclm* (**D**) and *Ho1* mRNA (**E**), in livers from WT and *Pask*^−/−^ mice. The value obtained in NON-FASTED WT mice was taken as 1. Bar graphs in (**A**–**C**) represent means ± SEM of the densitometric values normalized by total protein detected by Stain-Free (TOTAL PROTEIN) (Supplementary Figs S2 and S3); (**D**) and (**E**) represent means ± SEM, and the levels of expression were normalized by the mRNA of *β-Actin*; n = 4–5 animals per condition. *P < 0.05, ***P < 0.001 WT *vs. Pask*^−/−^; ^#^*P* < 0.05, ^##^*P* < 0.01, ^###^*P* < 0.001 NON-FASTED *vs*. FASTED.

The transcription factor NRF2 functions as a major regulator of the cellular redox balance^35^. Elevated levels of ROS induce NRF2 translocation to the nucleus and the transcription of its downstream regulated genes include GCLm (Glutamate-cysteine ligase modifier subunit) and HO1 (Heme Oxygenase 1). GCL is an enzyme that controls the rate-limiting step in the synthesis of GSH, a very powerful non-enzymatic endogenous antioxidant. HO1 catalyzes the first stage of the oxidation of heme to biliverdin-IXa, iron, and carbon monoxide. Accordingly, we checked NRF2, GCLm and HO1 protein expressions in the liver of PASK-deficient mice and shown that PASK deficiency altered the gene and protein expression of *Nrf2* (Figs 2D and 8A). Thus, under basal conditions, NRF2 protein expression was slightly lower in PASK-deficient mice. However, fasting decreased NRF2 protein levels in WT mice (Fig. 8A), although the gene expression of *Nrf2* was higher (Fig. 2D). In contrast, both *Nrf2* mRNA and NRF2 protein levels were upregulated when *Pask*^−/−^ mice were fasted. Similarly, lower levels of GCLm and HO1 proteins were detected in fasted WT mice, although *Glcm* and *Ho1* gene expression increased under this condition (Fig. 8B–E). As happened with NRF2, higher levels of *Gclm* and *Ho1* mRNAs and proteins were found in fasted PASK-deficient mice, compared to WT.

### ERK1/2 activation and PCNA protein were PASK dependent

**Figure 9:**
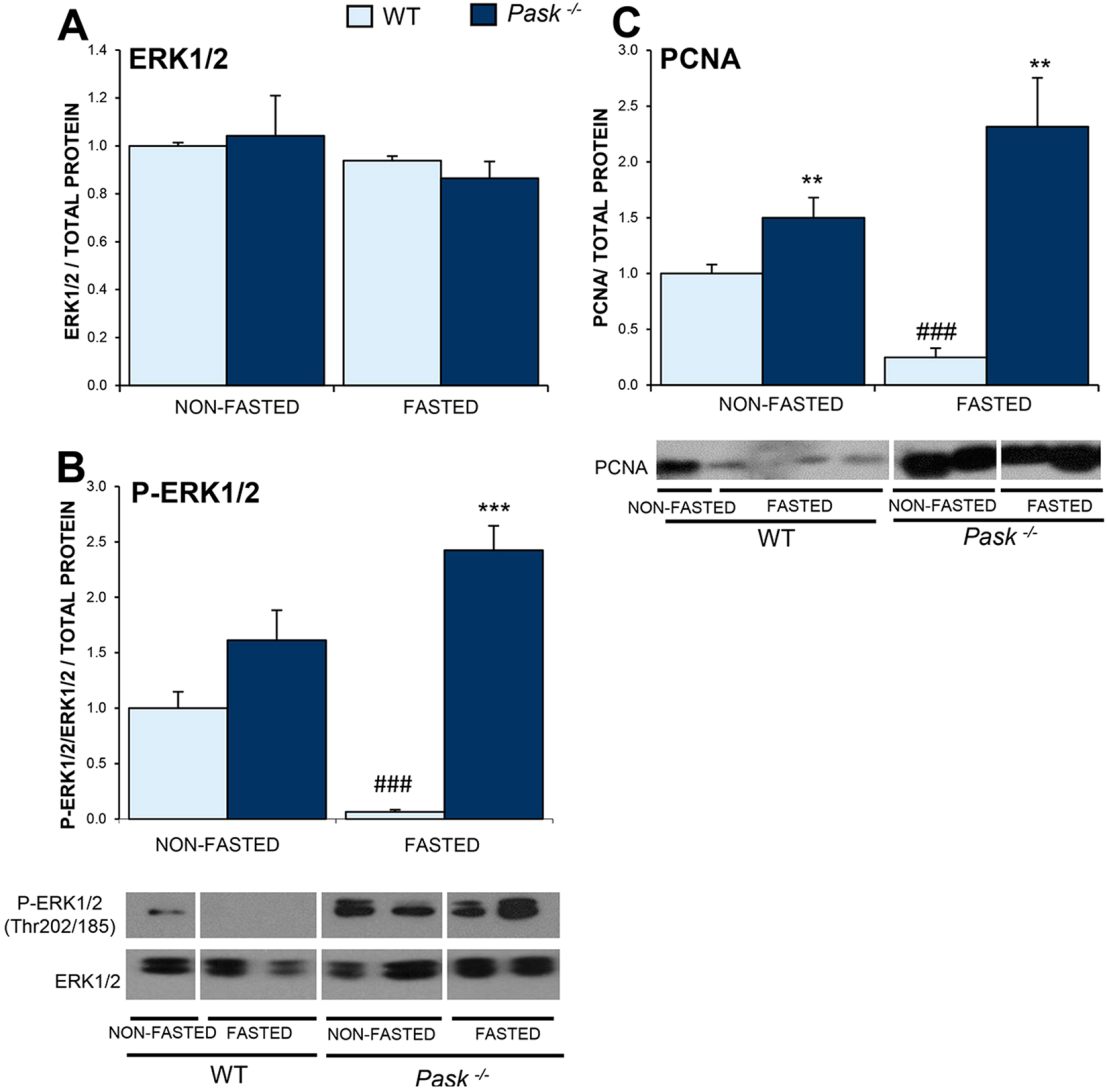
Effects of PASK deficiency on the ERK1/2 and PCNA proteins. Immunoblot analysis of total ERK1/2 (ERK1/2) (**A**), phospho-ERK1/2 (Thr202/185) (P-ERK1/2) (**B**) and PCNA (**C**), in livers from wild-type (WT) and PASK-deficient (*Pask*^−/−^) mice. Liver lysates under non-fasted (NON-FASTED) and fasted 48 h (FASTED) conditions were processed for Western blot analysis. The value obtained in NON-FASTED WT mice was taken as 1. Bar graphs represent means ± SEM of the densitometric values normalized by total protein detected by Stain-Free (TOTAL PROTEIN) (Supplementary Fig. S2); n = 4–5 animals per condition. **P < 0.01, ***P < 0.001 WT *vs. Pask*^−/−^; ^###^*P* < 0.001 NON-FASTED *vs*. FASTED.

The activation of ERK1/2 (extracellular signal-regulated protein kinases 1 and 2) might positively regulate the NRF2 pathway increasing nuclear translocation^36^. We checked the activation of this pathway, taking into account the ROS regulatory role in both pathways. Our results for WT mice indicated that prolonged fasting produced a complete fall in ERK1/2 activity and of cellular proliferation confirmed also by the downregulation of PCNA protein (proliferating cell nuclear antigen) (Fig. 9). These effects seemed to be PASK dependent, since fasting in PASK-deficient mice did not reduce ERK1/2 activity, keeping PCNA expression even higher than under basal conditions (Fig. 9B,C).

## Discussion

Redox status and oxidative stress are involved in inflammatory, metabolic and proliferative liver diseases. Oxidative stress is caused by dysfunctional mitochondrial oxidative phosphorylation and/or reduced antioxidant mechanisms^4^. It has been reported that an abnormal glucose metabolism, as occurs in diabetic or obese patients, increases levels of oxidative damage^37–39^.

PAS kinase (PASK) regulates glucose and energy metabolism homeostasis in response to metabolic requirements^24,40^. PAS domains can sense nutrients, intracellular oxygen, redox state and various metabolites^20,30,41^. Additionally, PASK-deficient mice are protected against obesity and the insulin resistance induced by high fat diets^23,26^.

The goal here was analyze the role that PASK plays in hepatic oxidative stress and the antioxidant response, considering its effects, as previously described, in hepatic *de novo* lipogenesis and glucose metabolism both in physiological and pathological (high fat diet) situations^23,25,26^, and that PASK deficiency regulates the AMPK and mTOR pathways^30^. Accordingly, we analyze the effect of PASK deficiency under normal and prolonged fasting conditions. This latter condition has been described as possibly promoting oxidative stress, although in other cases it protects against it^14,15,17,18,42^.

PASK deficiency has been associated with an elevated metabolic rate, also confirmed in PASK knockdown myoblast^23^ and neuroblastoma cells^21^. Accordingly, higher ATP content was found under fasted conditions in PASK-deficient liver and MEFs. This might be due to an increase in mitochondrial activity in PASK deficiency attributed to an increased mitochondrial content or function. To confirm the differences in mitochondrial activity or content, we have analyzed PCG1a, the main regulator of oxidative metabolism and mitochondrial biogenesis. PGC1a helps the cell adapt to conditions that require more energy, increasing mitochondrial mass^43–45^. PGC1a is a co-activator of several transcription factors (TF) and nuclear receptors for regulating different genes in the electron transport chain subunits^46^, Krebs cycle enzymes^46^, fatty acid oxidation^47^, and antioxidant components^48^. Previous data have not recorded any variations in both *Ppargc1a* and *Ppara* genes in HFD-fed PASK-deficient mice^23^. However, our data confirm that PASK deficiency alters the expression of *Ppargc1a* and several TFs and nuclear receptors of this pathway: *Nrf2*, *Ppara*, and *Pparg*. All of these TFs were overexpressed under fasted conditions, and *Ppargc1a* and *Pparg* also under basal conditions in *Pask*^−/−^ mice; suggesting that biogenesis may be stimulated in PASK-deficient mice and agree with the protection against obesity observed in these mice^23,25^. By contrast, previous reports have stated that the downregulation of PGC1a and its downstream cascade are associated with mitochondrial damage and decreased mitochondrial density in obesity^49^.

Post-transcriptional PGC1a activity is also regulated by deacetylation mediated by SIRT1^50^ and by phosphorylation through AMPK. Both activate PGC1a^51,52^, promoting its nuclear localization and inducing the expression of its target genes^53^. AMPK is also activated by fasting and energetic stress conditions to induce mitochondrial biogenesis and energy homeostasis^54–56^. However, we have previously described that hepatic AMPK and S6K1 activities were comparable in WT and PASK-deficient mice under fasting conditions^30^, suggesting that these proteins should not be responsible for the changes observed in *Ppargc1a* in PASK-deficient mice. But, like the PGC1a, the expression of *Sirt1* under basal conditions was higher in PASK-deficient mice, but this effect was not observed in the protein levels. SIRT1 also increased under fasted conditions, but no differences were found between the PASK-deficient and the WT mice. However, an analysis of the sub-cellular distribution indicates that SIRT1 was mainly located in the nucleus in PASK-deficient mice, in contrast to a cytosolic distribution in WT mice. This is consistent with what Gurd *et al*. have previously described, where an increase in nuclear SIRT1 activity positively correlates with the overexpression of genes regulated by PGC1a, although the protein amount was not modified^57^.

In addition, overexpression of mitochondrial DNA content and *Cox-IV* gene was found, with no significant changes in *Cs* in PASK-deficient mice. Our data, therefore, confirmed also by electron microscopy studies, did not show an increase in the mitochondrial average number, in agreement with previous data^23^. Nonetheless *Ppargc1a* and *Cox-IV* overexpression that we observed in PASK-deficient mice, have already been described^58,59^ as a marker of biogenesis. Also CS activity slightly increased with PASK deficiency suggesting an improvement in the efficiency of the mitochondrial function, as previously proposed^60^ although further assays are required to confirm this evidence.

Furthermore to biogenesis, cells maintain mitochondrial quality through dynamics mechanisms such as mitochondrial fusion and fission that respond to excess or deprivation of nutrients and concomitant cell stress. In this way, mitochondrial fusion in response to cell stress can protect from cell death and autophagy, while oxidative stress can trigger mitochondrial fission and cell death. In this sense, obesity and excess of nutrients intake is associated to accumulate damage and increased mitochondrial fission giving to fragmentation^61^. Damage mitochondrial can be selected to induce mitophagy by the presence of PINK1^62^ and BNIP3^63^. By contrast, mitochondrial fusion promoted in fasted conditions protects from mitophagy^64^. Our data showed that PASK deficiency increased gene expression of fusion proteins MFN1 (Mitofusin-1) and slight OPA1 in basal conditions. In accordance with that micrographs of liver sections from these mice showed slight more tubular mitochondria in contact to endoplasmic reticulum (ER), surprisingly in basal conditions, similar to those found in fasted WT mice. ER-mitochondria association is a physiological response to the adaptation of hepatic metabolism to nutritional cues (cellular mechanism to adapt to metabolic demand in fasting or low glucose availability)^65^.

Previous results have related elevated levels of hepatic MFN1 with enhanced insulin sensitivity and metabolic effects^66,67^. However, the opposite effect was also found by hepatic ablation of MFN1 in mice; they observed improved insulin sensitivity in a high-fat diet despite increased mitochondrial fragmentation, ROS production and lipid droplet size^68^. As expected, fasting induced gene overexpression of all fusion proteins MFN1, MFN2, OPA1 in the WT mice, in agreement to previous reports^11,66^. Likewise, in accordance to data found for biogenesis markers described here and other previous reports^67^. Additionally, a significantly induction of mitophagy marker BNIP3 was found in absence of FIS and PINK1, in accordance to previous reports that showed the overexpression of BNIP3 by fasting^63^. Likewise, they reported accumulated excess of ROS, hepatic lipid and steatohepatitis in livers of BNIP3 null mice^63^. Adaptation to nutrients availability depends in part of mobilization of lipid droplets. Starvation promotes lipolysis that supplies fatty acids to mitochondria^69^. Autophagy-lipophagy have been proposed as major regulators of hepatic lipid catabolism. Lipolysis by cytoplasmic lipases and efficient transfer of fatty acid to mitochondria requires mitochondrial fusion^70^ and promotes PPARa and PGC1a signaling to increase fatty acid oxidation and mitochondrial biogenesis^71^. Under fasted conditions expression of *Sirt1*, *Ppargc1a* and *Ppara* could be positive regulators of autophagy-lipophagy. However, enlarged mitochondria in tight contact to endoplasmic reticulum observed in electron micrographs of liver sections from fasted WT mice suggest fusion mitochondrial process that can protect of mitophagy as previously have been reported^64^. Similarly, the expression of *Mnf1*, *Mfn2* and *Bnip3* was stimulated in fasting conditions in PASK-deficient mice, although in this case correlated with lower *Opa1* expression and overexpression of *Fis1* and *Pink1*. Electron micrographs of liver sections showed both some enlarged mitochondria and a more fragmented pools, suggesting that in these conditions PASK deficiency might increase mitophagy and mitochondria remodeling (more fusion and fission at the same time) suggesting an active response against adverse situations. An increased number of lipid droplets were also observed in some hepatocytes from fasted PASK-deficient mice, that might be in accordance to previous reports describing that fatty acid mobilization by autophagy was accompanied of moving of free fatty acid to lipid droplets to avoid toxicity of excess of cytosolic lipids in organelles membrane integrity^70^. In this sense, we have also reported that *Cpt1a* expression (essential for fatty acid transport and oxidation to obtain energy) under fasting conditions were significant lower in PASK-deficient mice^26^. Our results suggest that PASK modulates mitochondrial dynamics, although more detailed studies are necessary to clarify its function in that process.

Mitophagy has been associated to FOXO3a transcription factor, that controls PINK1 expression^72^. Under fasting conditions, elevated SIRT1 and higher AMPK activity promote FoxO3a transcriptional activity and nuclear translocation^73,74^. An interesting *datum* is that PASK deficiency overexpressed *FoxO3a* under both basal and fasting conditions. All-in-all, we show that PASK deficiency under fasting conditions increases nuclear location of SIRT1 and AMPK activation^30^, possibly to induce FoxO3a translocation to the nucleus. This translocation could facilitate the expression of antioxidant genes containing FoxO3a binding elements.

Mitochondria mainly generate ROS, which can damage DNA, lipids and proteins, however, cells contain antioxidant enzymes to protect themselves. PGC1a has been reported to induce the expression of antioxidant enzymes like SOD and GPx^48,75,76^. Our data show that PASK-deficient MEFs are better protected against a stress situation, as they are able to block the H_2_O_2_ stimulated increase in ROS. Nevertheless, high ROS levels even under standard culture conditions in PASK-deficient MEFs decreased proliferation rate compared to WT (data not show). Our results also show that PASK-deficient mice significantly increase the expression of hepatic antioxidant enzyme genes in response to fasting (*MnSod*, *Cu/ZnSod* and *Gpx*), and slightly increase the *Cat* gene. These results are consistent with the observed overexpression of *Ppargc1a*, *Sirt1* and *FoxO3a*. Furthermore, in the case of *Gpx* and *MnSod* PASK-deficient mice also recorded higher expression levels in basal state, in addition to fasting. Also, a slight higher MnSOD activity was detected in basal and under 24 h fasting in PASK-deficient mice, although this effect was not maintained in prolonged fasting. Nevertheless, a reduction in ROS levels were recorded even under basal conditions. Previous reports have shown that mice overexpressing MnSOD are partially protected against the insulin resistance induced by a high-fat diet^77^, preventing the obesity triggered by glucose intolerance^78^, as happened with PASK-deficient mice^23,26^.

Additionally, PGC1a activation by SIRT1 could lead to NRF2 transcriptional activity^79^. The transcription factor NRF2 is the major regulator of the cellular redox balance^35^. Under physiological conditions, NRF2 is permanently degraded by the proteasome. However, in response to an increase in oxidative stress, NRF2 avoids its degradation and translocate into the nucleus, where induces the expression of the *Gclm* and *Ho1* genes among others^80^. Our results indicated that PASK deficiency overexpressed proteins and mRNA of *Nrf2*, *Gclm* and *Ho1* under fasting conditions. However, we observed a discordance between the protein levels and mRNA of these genes in fasted WT mice. This could be due to the complex mechanism of functional regulation of NRF2, such as, for example, proteasome-dependent degradation by KEAP1(Kelch-like ECH-associated protein 1) -NRF2 interaction or others post-transcriptional mechanisms.

Likewise, NRF2 activation could be regulate positively by phosphorylation^36,81^. Here, we show that PASK deficiency promoted ERK1/2 over-activation and previously also showed an over-activated PI3K-Akt pathway in PASK-deficient mice^22,26^. The higher activation of ERK1/2 and elevated PCNA levels were observed under fasted conditions in PASK-deficient mice, in accordance with the increased expression of *Gclm* and *Ho1* as a NRF2 target genes. The simultaneous activation of ERK1/2 and the high level of PCNA suggest a more regenerative state, but it should be noted that this could respond to previous damage^82^. However, additional studies are needed to confirm this suggestion.

## Conclusions

In sum, PASK deficiency, under basal conditions, increased the expression of coactivator *Ppargc1a*, transcription factors such as *Pparg and FoxO3a*, and activators such as deacetylase *Sirt1*, all of which are involved in the regulation of oxidative metabolism and mitochondrial biogenesis. Although PGC1a and SIRT1 protein levels were not modified, SIRT1 location was mainly nuclear. The stimulation of this pathway was observed at the level of the overexpression of the ROS-detoxifying enzymes MnSOD and GPx and confirmed by a lower production of ROS. Likewise, mitochondrial elevated levels of MFN1 fusion proteins that should maintain mitochondrial integrity in PASK-deficient mice. Additionally, under prolonged fasting conditions, PASK deficiency improved the expression of: the antioxidant enzymes (*MnSod*, *Cu/ZnSod*, *Gpx*, *Gclm* and *Ho1)*, the transcription factor *FoxO3a* and PINK1 involved in cell survival and mitophagy respectively. Additionally, stimulated mitochondrial biogenesis through overexpressing *Ppargc1a*, *Ppara*, *Pparg* and *Nrf2*. Thus, although COX-IV levels and ATP production were higher under fasting conditions than in WT, ROS levels remain controlled, and the overactivation of the MAPK pathway seemed to maintain a regenerative state. All these effects of PASK deficiency are interesting for states that promote an increase in oxidative stress, such as aging, diabetes, obesity and others. Here we have described new evidence in this field, whereby PASK blocking is a powerful promotor of antioxidant mechanisms against oxidative stress in the liver.

## Electronic supplementary material

Supplementary Information
