# Supplementary material for: Pas Kinase Deficiency Triggers Antioxidant Mechanisms in the Liver
Source: Sci Rep. 2018 Sep 14;8:13810. doi: 10.1038/s41598-018-32192-w (PMC6138710; doi:10.1038/s41598-018-32192-w)
Supplement: Supplementary file 1 — Supplementary Information [file 41598_2018_32192_MOESM1_ESM.pdf]

PAS KINASE DEFICIENCY TRIGGERS ANTIOXIDANT MECHANISMS IN THE LIVER

Dongil P[1,3$], Perez-Garcia A[1,3$], Hurtado-Carneiro V[1,2], Herrero-de-Dios C[3], Blazquez E[1,2], Alvarez E[1,2#] and Sanz C[2,3#*]

$ These authors have contributed equally.

[#] These authors have contributed equally to this work

[1]Department of Biochemistry and Molecular Biology, Faculty of Medicine, Complutense University of Madrid, Institute of Medical Research at the Hospital Clínico San Carlos (IdISSC), Ciudad Universitaria, s/n, 28040 Madrid, Spain. [2]Spanish Biomedical Research Centre in Diabetes and Associated Metabolic Disorders (CIBERDEM), Spain, URL www.ciberdem.org. [3]Department of Cell Biology, Faculty of Medicine, Complutense University of Madrid, Spain

*Corresponding author: Carmen Sanz. Departamento de Biología Celular, Facultad de Medicina, Universidad Complutense, Plaza S. Ramón y Cajal, s/n, 28040-Madrid, Spain. Fax. +34 913 941 691, e-mail: mcsanz@med.ucm.es (C. Sanz)

Running Title: PAS kinase and hepatic oxidative stress

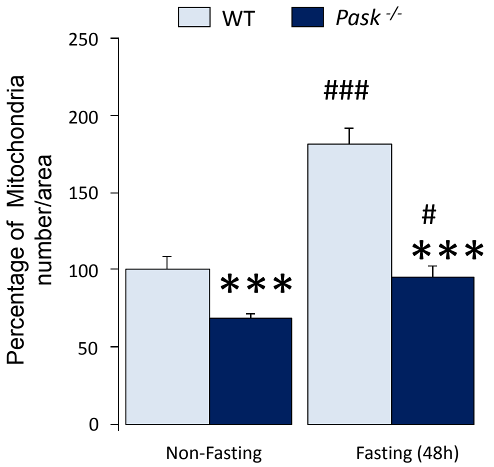

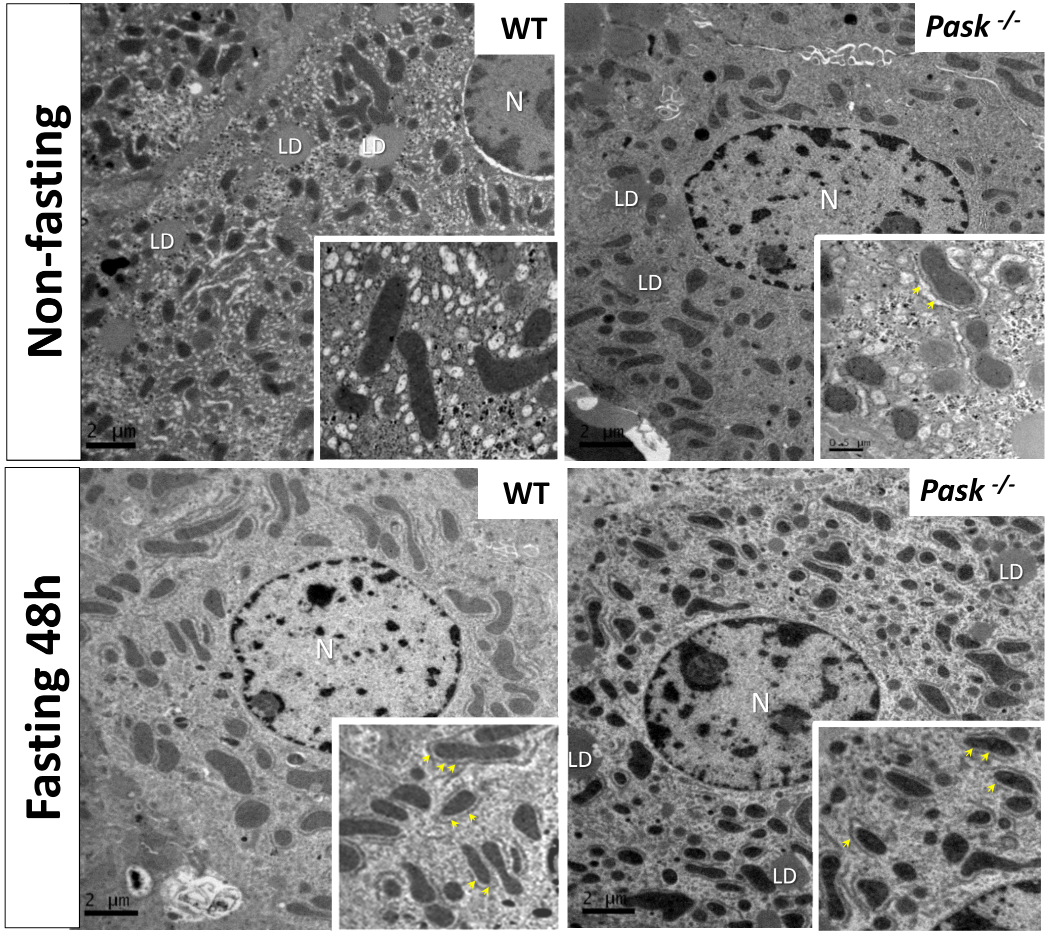

Figure S1. Mitochondrial quantification and morphology from liver slices of WT and Pask-deficient mice. Tthe mitochondria were visualized by a JEOL JEM 1010 transmission electronic microscope (TEM) and micrographs were taken at 6K magnification. Mitochondrial counts were performance at least from 7 different hepatocytes per genotype. The data presented were the average of mitochondrial number±S.E.M, expressing them all as a percentage (taking 100 % the quantity in WT non-fasting) and were normalized by total area (µm2). *** $p<0.001$ WT *vs* *Pask*$^{-/-}$; # $p< 0.05$, ### $p<0.001$ non-fasting *vs* fasting. Micrographs (6000x) are representative examples of data. Inserts show higher magnification for details of mitochondria morphology. Arrows indicate RER (rough endoplasmic reticulum)-mitochondria association. N: nucleus, LD: lipid drop.

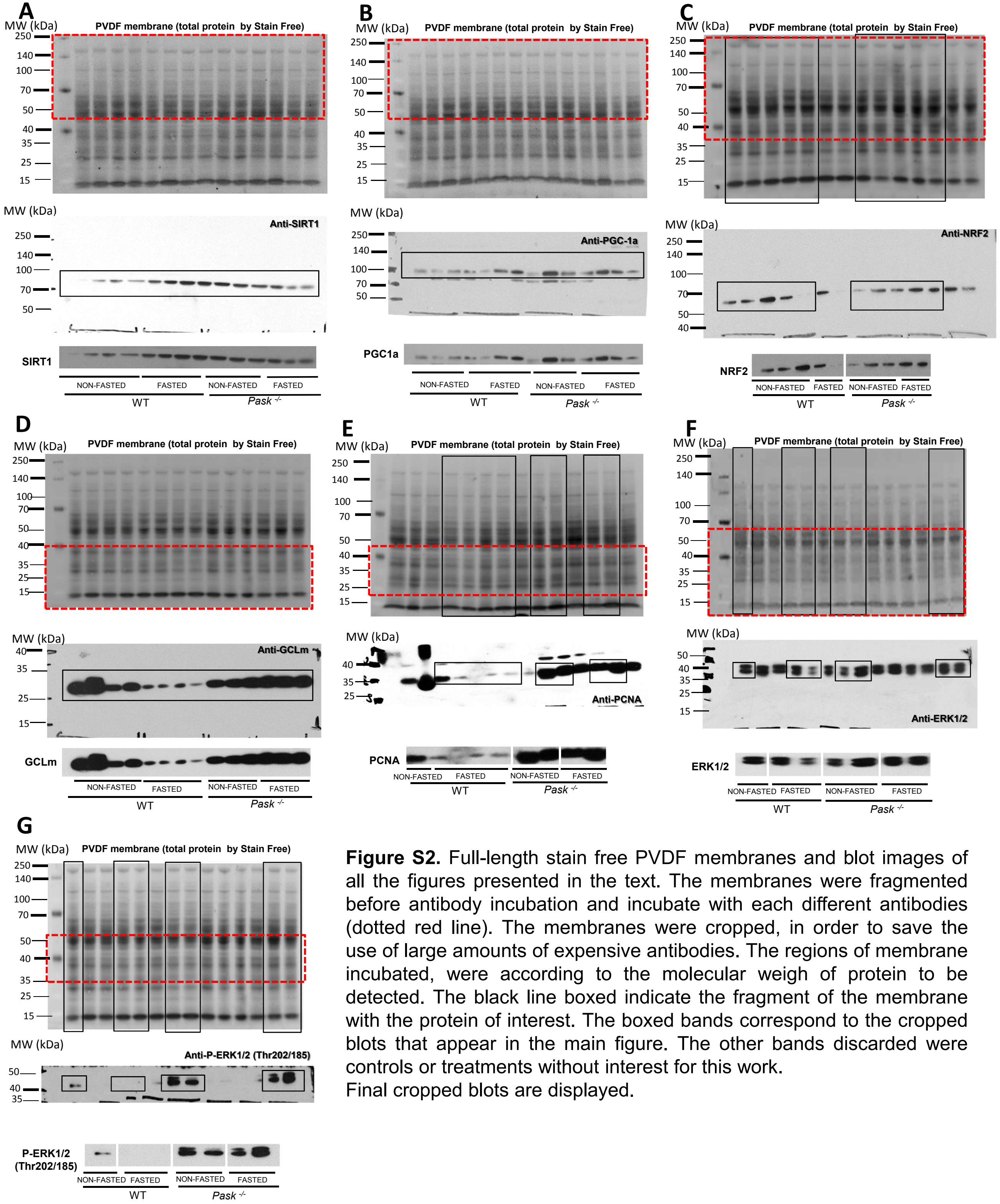

Figure S2. Full-length stain free PVDF membranes and blot images of all the figures presented in the text. The membranes were fragmented before antibody incubation and incubate with each different antibodies (dotted red line). The membranes were cropped, in order to save the use of large amounts of expensive antibodies. The regions of membrane incubated, were according to the molecular weigh of protein to be detected. The black line boxed indicate the fragment of the membrane with the protein of interest. The boxed bands correspond to the cropped blots that appear in the main figure. The other bands discarded were controls or treatments without interest for this work.
Final cropped blots are displayed.

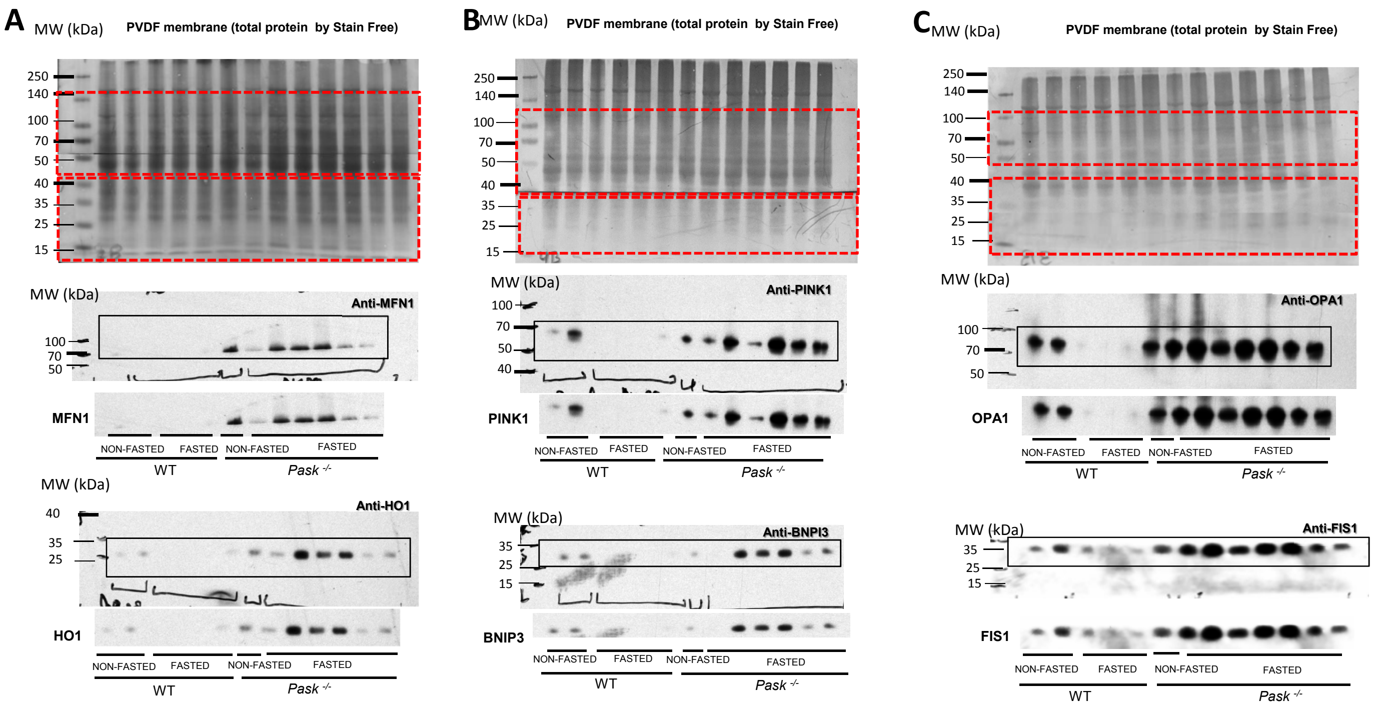

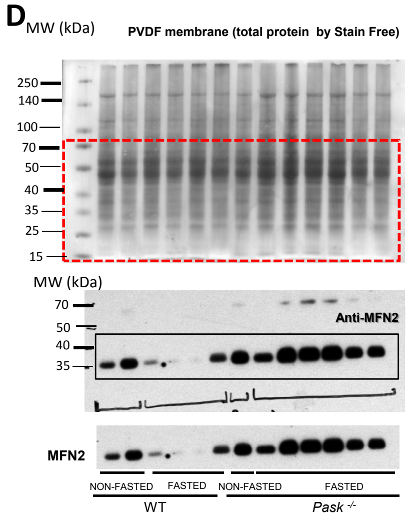

Figure S3. Full-length stain free PVDF membranes and blot images of all the figures presented in the text. The membranes were fragmented before antibody incubation and incubate with each different antibodies (dotted red line). The membranes were cropped, in order to save the use of large amounts of expensive antibodies. The regions of membrane incubated, were according to the molecular weigh of protein to be detected. The boxed (black lines) indicate the fragment of the membrane with the protein of interest, displayed in the main figures.

Supplementary Table 1. Identification of primers used in the different gene expression assays.

REAL-TIME POLYMERASE CHAIN REACTION (SYBR GREEN qRT-PCR)

**Gene (Protein)**	**Mouse Forward primer**	**Mouse Reverse primer**
12S rRNA (12S) (mitochondrial)	5’-CAAACTGGGATTAGATACCCCAC-3’	5’-GAGGGTGACGGGCGGTGTGT-3’
Actb (β-ACTIN)	5’-CTCTCTTCCAGCCTTCCTTC-3’	5’-GGTCTTTACGGATGTCAACG-3’
Bnip3 (BNIP3)	5’-CAGCATGAATCTGGACGAAG-3’	5’-ATCTTCCTCAGACAGAGTGC-3’
Cat (CAT)	5’-GAATGGCTATGGTCCACACA-3’	5’-CAAGTTTTTGATGCCCTGGT-3’
CoxIV (COXIV)	5’-TGAATGGAAGACAGTTGTGGG-3’	5’-GATCGAAAGTATGAGGGATGGG-3’
Cs (CS)	5’-GGGACTTGTGTATGAGACTTCG-3’	5’-AGCCAAAATAAGCCCTCAGG-3’
Cu/ZnSod (Cu/ZnSOD)	5’-GGTGGTCCACGAGAAACAAG-3’	5’-CAATCACACCACAAGCCAAG-3’
Fis1 (FIS1)	5’-GCCCCTGCTACTGGACCAT-3’	5’-CCCTGAAAGCCTCACACTAAGG-3’
FoxO3a (FoxO3a)	5’-TACGAGTGGATGGTGCGCTG-3’	5’-AGGTTGTGCCGGATGGAGTTC-3’
Gclm (GCLm)	5’-TGTGTGATGCCACCAGATTT-3’	5’- GATGATTCCCCTGCTCTTCA-3’
Gpx (GPx)	5’-TGCAATCAGTTCGGACATC-3’	5’-CACCTCGCACTTCTCAAACA-3’
Ho1 (HO1)	5’-AGCCCCACCAAGTTCAAACA-3’	5’-CATCACCTGCAGCTCCTCCA-3’
Mcad (MCAD)	5′-TTCGAAGACGTCAGAGTGC-3′	5′-GCGACTGTAGGTCTGGTTC-3′
Mfn1 (MFN1)	5’-TCTCCAAGCCCAACATCTTCA-3’	5’-ACTCCGGCTCCGAAGCA-3’
Mfn2 (MFN2)	5’-AGGAGTGGTGTGGAAGGCAG-3’	5’-ACAAACTGGCGCTTGAAGG-3’
MnSod (MnSOD)	5’-AAGGAGCAAGGTCGCTTACA-3’	5’-ACACATCAATCCCCAGCAGT-3’
Nrf2 (NRF2)	5’-CTACTCCCAGGTTGCCCACA-3’	5’-CGACTCATGGTCATCTACAAATGG-3’
Opa1 (OPA1)	5’-CTGGAAGAATCGGACCCAAG-3’	5’-AGGTTCTTCCGGACTGTGGT-3’
Pink1 (PINK1)	5’-CTACCGCTTCTTCCGCCAGT -3’	5’-AGCCCGAAGGCCAGAAAGAC-3’
Ppara (PPARa)	5’-TGTTTGTGGCTGCTATAATTT-3’	5’-GCAACTTCTCAATGTAGCCTA-3’
Pparg (PPARg)	5’-GTGCCAGTTTGCATCCGTAGA-3’	5’-GGCCAGCATCGTGTAGATGA-3’
Ppargc1a (PGC1a)	5’-ATGTGTCGCCTTCTTGCTCT-3’	5’-ATCTACTGCCTGGGGACCTT-3’
Sirt1 (SIRT1)	5’-TTGTGAAGCTGTTCGTGGAG-3’	5’-GGCGTGGAGGTTTTTCAGTA-3’

Supplementary Table 2. Antibodies and conditions used for western blot assays.

Antibody	Host	Manufacturer	Dilution used
Anti-BNIP3	Mouse	Santa Cruz Biotechnology, California, USA	1:1000
Anti-ERK1/2	Rabbit	Millipore Iberica, Madrid, Spain	1: 2000
Anti-FIS	Mouse	Santa Cruz Biotechnology, California, USA	1:1000
Anti-GCLm	Rabbit	Sigma Aldrich, Madrid, Spain	1: 5000
Anti-HO1	Mouse	Santa Cruz Biotechnology, California, USA	1:1000
Anti-MFN1e	Mouse	Santa Cruz Biotechnology, California, USA	1:1000
Anti-MFN2	Mouse	Santa Cruz Biotechnology, California, USA	1:1000
Anti-NRF2	Rabbit	GeneTex, Irvine, EEUU	1: 1000
Anti-OPA1	Mouse	Santa Cruz Biotechnology, California, USA	1:1000
Anti-PCNA	Mouse	Abcam, Cambrigde, United Kingdom	1: 1000
Anti-PGC1a	Rabbit	Milipore Iberica, Madrid, Spain	1: 1000
Anti-phospho ERK1/2 (Thr202/185)	Mouse	Cell Signaling Technology, Danvers, MA, USA	1: 1000
Anti-PINK1	Mouse	Santa Cruz Biotechnology, California, USA	1:1000
Anti-SIRT1	Rabbit	Santa Cruz Biotechnology, California, USA	1: 500
Anti-Rabbit-HRP	Goat	Milipore Iberica, Madrid, Spain	1: 5000
Anti-Mouse-HRP	Goat	Bethyl Laboratories, Montgomery, USA	1: 5000
